# Wogonin Induced Calreticulin/Annexin A1 Exposure Dictates the Immunogenicity of Cancer Cells in a PERK/AKT Dependent Manner

**DOI:** 10.1371/journal.pone.0050811

**Published:** 2012-12-12

**Authors:** Yong Yang, Xian-Jing Li, Zhen Chen, Xuan-Xuan Zhu, Jing Wang, Lin-bo Zhang, Lei Qiang, Yan-jun Ma, Zhi-yu Li, Qing-Long Guo, Qi-Dong You

**Affiliations:** 1 State Key Laboratory of Natural Medicines, China Pharmaceutical University, Nanjing, People's Republic of China; 2 Department of Pharmacology, Jiangsu Provincial Hospital of Traditional Chinese Medicine, Nanjing, People's Republic of China; University of Frankfurt - University Hospital Frankfurt, Germany

## Abstract

In response to ionizing irradiation and certain chemotherapeutic agents, dying tumor cells elicit a potent anticancer immune response. However, the potential effect of wogonin (5,7-dihydroxy-8-methoxyflavone) on cancer immunogenicity has not been studied. Here we demonstrated for the first time that wogonin elicits a potent antitumor immunity effect by inducing the translocation of calreticulin (CRT) and Annexin A1 to cell plasma membrane as well as the release of high-mobility group protein 1 (HMGB1) and ATP. Signal pathways involved in this process were studied. We found that wogonin-induced reactive oxygen species (ROS) production causes an endoplasmic reticulum (ER) stress response, including the phosphorylation of PERK (PKR-like endoplasmic reticulum kinase)/PKR (protein kinase R) and eIF2α (eukaryotic initiation factor 2α), which served as upstream signal for the activation of phosphoinositide 3-kinase (PI3K)/AKT, inducing calreticulin (CRT)/Annexin A1 cell membrane translocation. P22/CHP, a Ca^2+^-binding protein, was associated with CRT and was required for CRT translocation to cell membrane. The releases of HMGB1 and ATP from wogonin treated MFC cells, alone or together with other possible factors, activated dendritic cells and induced cytokine releases. In vivo study confirmed that immunization with wogonin-pretreated tumor cells vaccination significantly inhibited homoplastic grafted gastric tumor growth in mice and a possible inflammatory response was involved. In conclusion, the activation of PI3K pathway elicited by ER stress induced CRT/Annexin A1 translocation (“eat me” signal) and HMGB1 release, mediating wogonin-induced immunity of tumor cell vaccine. This indicated that wogonin is a novel effective candidate of immunotherapy against gastric tumor.

## Introduction

Traditional cancer treatment methods include surgery, radiation therapy, chemotherapy, and for some cancer types, hormone therapy. Although the benefits are obtained by many patients, they are rarely curative for the very few residual disseminated tumor cells, the primary cause of death among cancer patients. An important reason why tumors are not controlled by the immune system is that the low immunogenicity. The use of cancer vaccines to elicit a therapeutic antitumor immune response against judiciously chosen tumor antigens expressed in the tumor cells can seek out and kill the disseminated tumor cells. One possible strategy for achieving this involves immunization with tumor cells that have been treated with a particular class of chemotherapeutic drugs.

Accumulating evidence indicates that several chemotherapeutic agents (including anthracyclines and oxaliplatin), and ionizing irradiation (such as γ-rays and ultraviolet C (UVC) light) induce immunogenic cancer cell death [1], [2]. It was suggested that they have capacity to drive calreticulin (CRT) translocation to the tumor cell surface, which acts as an “eat me” signal, is identified by dendritic cells (DCs), resulting in antitumor T-cell response [3]. The elements of the pathway mediating pre-apoptotic CRT exposure involve a pool of CRT that transited the Golgi apparatus and secreted by SNARE-dependent exocytosis [4].

HMGB1 (high-mobility group protein 1), a nuclear protein that is released from dying cell, is the ligand of Toll-like receptor 4 (TLR4) [1]. Depletion of HMGB1 from dying tumor cells abolishes the TLR4-dependent, DC-mediated presentation of antigens from dying tumor cells in vitro and in vivo [1]. So, HMGB1 release is required for the immunogenicity of cell death through its effect on TLR4. However, neither HMGB1 nor CRT (nor a combination of both) can promote complete DCs maturation, indicating that the search for immune-stimulatory molecules produced by dying cells must be continued [5].

Wogonin (5,7-dihydroxy-8-methoxyflavone), an active component isolated from *Scutellaria baicalensis* radix, was reported having significant anticancer activities by inducing cell differentiation, apoptosis and cell cycle arrest [6]–[8]. In this study, we tested whether wogonin, like some chemotherapy drugs mentioned above, is able to induce immunogenic cancer cell death, and if so, the possible signal pathways involved in this process were evaluated. We found for the first time that wogonin elicits a potent antitumor immunity effect by inducing the translocation of CRT and Annexin A1 to cell plasma membrane, as well as release of HMGB1 and ATP. We found that Endoplasmic Reticulum (ER) stress response, including PERK (PKR-like endoplasmic reticulum kinase)/PKR (protein kinase R) and eIF2α (eukaryotic initiation factor 2α subunit) phosphorylation and the subsequent activation of PI3K/AKT signaling pathway are involve in this process.

## Materials and Methods

### Ethics Statement

All animals were maintained in specific pathogen-free conditions, and all experiments were carried out according to the Federation of European Laboratory Animal Science Association guidelines. The Ethics Committee of China Pharmaceutical University approved all the animal experiments (Permit numbers: SYXK2007-0025).

### Chemicals and reagents

Wogonin was applied in DMSO to 10 mM and stored at −20°C. Doxorubicin, Rapamycin, LY294002, AKT inhibitor X (AKTi) were purchased from CalbioChem (San Diego, CA). EGFR, ERK1/2, AKT1/2, Ku 80, goat anti-rabbit IgG-HRP and goat anti-mouse IgG-HRP antibody were purchased from Santa Cruz Biotechnology (Santa Cruz, CA). N-Acetyl-Cysteine (NAC) and monoclonal mouse anti-β-actin were obtained from Sigma (St. Louis, MO). p-PERK (Thr980), PERK, p-eIF2-α (Ser51), eIF2-α, p-PKR (Thr446/451), PKR, p-AKT (Ser 473), p-AKT (Thr 308), Annexin A1, p-S6K (Thr389), p-4E-BP1 (Ser 65), S6K, 4E-BP1, p-GSK3β(Ser 9), p-S6 (S235/236), p-ERK (Thr202/Tyr204), p-JNK (Thr183/Tyr185) and p-p38(Thr 180/Tyr182) antibodies were purchased from Cell Signaling Technology (Bevery, MA).

### Cells culture

Primary cultured mouse monocyte-derived dendritic cells (MoDCs) were from B6 mice bone marrow, maintained in a MEM medium (Sigma, St. Louis, MO) supplemented with a 10% FBS plus GM-CSF (50 ng/ml). For Western blot analysis, cells were reseeded in 6-well plates at a density of 2×10^5^ cells/ml with fresh complete culture medium. The human gastric carcinoma MKN-45 cells, mouse gastric carcinoma MFC cells derived from 615 mice (Military Medical Sciences, Beijing), WT and AKT1/2 Double Knockout MEFs (purchased from Shanghai cell bank, Chinese Academy of Sciences, Shanghai, China)were maintained in a DMEM medium (Sigma, St. Louis, MO), supplemented with a 10% fetal bovine serum (Invitrogen, Carlsbad, CA), Penicillin/Streptomycin (1∶100, Sigma, St. Louis, MO) and 4 mM L-glutamine (Sigma, St. Louis, MO), in a CO_2_ incubator at 37°C.

### Two-dimensional gel electrophoresis and protein identification by mass spectrometry

The methods were referenced to the previous study [9]. Briefly, Isoelectric focusing (IEF) was performed on an Ettan IPGphor II (Amersham Bioscience) with 24-cm immobilized pH gradient strips (pH 3–10; Amersham Bioscience). Gels (three repetitions of each) were silver-stained according to published procedures and scanned using an Atrix scan 1010 plus (Microtek, Taiwan, China), and resulting images were analyzed using the ImageMaster 2D Platinum software (Amersham Bioscience) for spot detection, quantification, and comparative and statistical analyses. Detected spots and four control spots in nonstained gel areas were excised (approximately 1-mm^3^ cubes) from the gels stained with Coomassie Brilliant Blue. Sample preparation for matrix-assisted laser desorption/ionization time-of-flight (MALDI-TOF).

### Double or Triple-Label Immunofluorescence Confocal Microscopy

MFC cells were fixed with 4% paraformaldehyde and processed for immunofluorescence, as described previously [10], except that Cy5-, fluorescein isothiocyanate- and/or tetramethylrhodamine B isothiocyanate-labeled secondary antibodies (Jackson ImmunoResearch Laboratories, West Grove, PA) were used. Then, cells were washed briefly in PBS and mounted on glass slides by using ProLong antifade kit mounting reagent (Molecular Probes). Cells were visualized with an Axiovert S100 inverted microscope (Carl Zeiss) coupled to a CARV confocal unit (Atto Bioscience, Rockville, MD) and Hg vapor light source. Images were collected using Openlab software version 3.0.9 (Improvision, Lexington, MA) with an ORCA-ER digital camera (Hamamatsu Photonics, Hamamatsu City, Japan) and filter sets for fluorescein isothiocyanate (484 nm), tetramethylrhodamine B isothiocyanate (555 nm), and Cy5 (650). Image analysis of confocal images was performed using Adobe Photoshop 5.5. To allow intensity comparisons, we used similar conditions to collect and manipulate images within each experiment.

### Western blots

As mentioned before [11], 30 µg of protein from each indicated treatments was separated by 10% SDS–polyacrylamide gel electrophoresis (SDS-PAGE) and transferred onto a polyvinylidene difluoride (PVDF) membrane (Millipore, Bedford, MA). After blocking with 10% milk for 30 min, membranes were incubated with primary antibodies overnight at 4°C followed by incubation with secondary antibodies for 1 hour at room temperature. Antibody binding was detected with the enhanced chemiluminescence (ECL) detection system. Western blots results were quantified by Image J software.

### Biotinylation of MFC cell surface proteins

Biotinylation and recovery of cell surface proteins were performed with a method adapted from reference [1]. Briefly, 15×10^6^ MFC cells were placed on ice and washed three times with ice-cold PBS-Ca^2+^-Mg^2+^(PBS with 0.1 mM CaCl_2_ and 1 mM MgCl_2_). Membrane proteins were then biotinylated by a 30-min incubation at 4°C with NHS-SS-biotin 1.5 mg/ml (Pierce) freshly diluted into biotinylation buffer (10 mM triethanolamine, 2 mM CaCl_2_, 150 mM NaCl, pH 7.5) with gentle agitation. MFC cells were rinsed with PBS-Ca^2+^-Mg^2+^ + glycine (100 mM) and washed in this buffer for 20 min at 4°C to quench unreacted biotin. The cells were then rinsed twice with PBS-Ca^2+^-Mg^2+^, scraped in cold PBS, and pelleted at 3,000 rpm at 4°C. The pellets were solubilized for 45 min in 600 µl of lysis buffer (1% Triton X-100, 150 mM NaCl, 5 mM EDTA, 50 mM Tris, pH 7.5) containing protease inhibitors. The lysates were clarified by centrifugation at 14,000× g for 10 min at 4°C, and the supernatants were incubated overnight with packed streptavidin-agarose beads to recover biotinylated proteins. The beads were then pelleted by centrifugation, and aliquots of supernatants were taken to represent the unbound, intracellular pool of proteins. Biotinylated proteins were eluted from the beads by heating to 100°C for 5 min in SDS-PAGE sample buffer before loading onto a 10% SDS-PAGE gel. To ensure the absence of leakage of biotin into the cells, we systematically verified the absence of the intracellular protein actin in biotinylated extracts.

### Immunoprecipitation (IP)

As mentioned before [11], cells treated with the appropriate stimuli were lysed with lysis buffer, 200 mM NaCl (pH 7.4), 1% Triton X-100, 10% glycerol, 0.3 mM EDTA, 0.2 mM Na3VO4, and protease inhibitor cocktails (Roche Diagnostics, Indianapolis, IN). Aliquots of 600 µg of proteins from each sample were precleared by incubation with 20 µl of protein A/G Sepharose (beads) (Amersham, IL) for 1 hour at 4°C. Precleared samples were incubated with anti-Calreticulin (CRT) Antibody (cs-2891, Cell Signaling Tech), or anti-DNA-PKcs (sc-9051, Santa Cruz antibodies) in lysis buffer overnight at 4°C. 30 µl of protein A/G beads were added and the samples were incubated for 2 hours at 4°C. The beads were washed five times with phosphate-buffered saline (PBS) and once with lysis buffer, boiled, separated by 10% SDS-PAGE, and transferred onto a PVDF membrane followed by Western blotting analysis as described above.

### CRT-Confocal immune-fluorescence

Cancer cells with indicated treatment were fixed and blocked with 10% BSA in PBS for 30 min at room temperature and then incubated with 1∶100 rabbit anti-calreticulin (CRT) Antibody (cs-2891, Cell Signaling Tech) for 1 h(testing CRT translocation) followed by FITC–anti-rabbit secondary antibody (Cell Signaling Tech, MA) at 1∶100 for 30 min and CRT immune-fluorescence was observed in a confocal microscopy(Leica TCS SMD FCS, Leica, Germany), Hoechst 33342 was used to stain the nuclear.

### Small interfering RNA (siRNA) knockdown studies

As described before [11], siRNA for PERK (sc-36214), DNA-PKcs (sc-35200) or p22 (sc-142330) was purchased from Santa Cruz Biotechnology (Santa Cruz, CA). Cancer cells were cultured in a complete medium that did not contain antibiotics for 4 days. Cells were seeded in a 6-well plate 1 day prior to transfection and cultured to 60% confluence the following day. For RNAi experiments, 6.25 µl of Lipofectamine™ LTX together 2.5 µl PLUS™ Reagent (Invitrogen, Carlsbad, CA) was diluted in 90 µl of DMEM for 5 min in room temperature. Then, 10 µl of siRNA (20 µM) was mixed with DMEM containing Lipofectamine together with PLUS reagent and incubated for 30 minutes at room temperature for complex formation. Finally, the complex was added to the wells containing 2 ml medium with 100 nM final siRNA concentration. P22 (ab56953, abcam) or PERK (sc-13073, Santa Cruz) protein expression was determined by Western blot 48 hours after transfection.

### Reactive Oxygen Species (ROS) detection

As described before [11], cancer cells were pre-loaded with 1 µM of fluorescent dye dihydrorhodamine (DHR) (Invitrogen, Carlsbad, CA) two hours before indicated treatments, which react with ROS in cells and results in a change of fluorescence. After being treated with indicated treatments, cancer cells were trypsinized, suspended in ice-cold PBS and fixed in 70% ethyl alcohol in −20°C. The changes in fluorescence in drug-treated cells were quantified by FACS analysis. Induction of ROS generation was expressed in arbitrary units. ROS production was also detected by visualizing dihydrorhodamine (DHR) fluorescence under confocal immune-fluorescence microscopy.

### Measurement of extracellular ATP levels

ATP synthesis was measured in MFC cells under the various treatment of wogonin. In sextuplicates, 5×10^3^ cells were cultured in each well of a 96-well plate. After incubation at 37°C for 24 h, the amount of ATP in the supernatant was measured using the Molecular Probes' ATP Determination Kit (Kaiji,Nanjing) according to the manufacturer's directions. This kit is based on the bioluminescence detection of ATP, using recombinant firefly luciferase and its substrate, luciferin. Total chemiluminescence was collected by a luminometer. The amount of ATP from a test solution was quantified by comparison to a calibration curve using ATP as the standard.

### Cytokine Quantification

MFC cancer cells were treated with indicated treatments for 36 hours; supernatant samples were collected and assessed for HMGB1 using ELISA kits (Shino-Test Corporation, ST51011) according to its protocol. For cytokine release, MoDCs were treated with supernatant of MFC cells for 24 hours, supernatant of MoDCs were collected and assessed for IL-6 (Mouse IL-6 ELISA Kit, BD OptEIA, 550950) and TNF-α (Mouse TNF-α ELISA Kit, BD OptEIA, 560478) according to their protocols.

### In vivo anti-tumor cell vaccination experiment

3×10^6^ MFC cells, suspended in 200 ml PBS, either left untreated or treated with wogonin (100 µM) for 4 h. Wogonin treated MFC cells were inoculated subcutaneously into the lower flank of 6-week-old female 615 mice (Military Medical Sciences, Beijing), whereas 5×10^5^ untreated cells were inoculated into the contralateral flank 7 days later as described in previous study [1].

### Phagocytosis assay

The phagocytosis assay was performed as previously described [12]. MoDCs were adjusted to 2.5×10^4^cells/ml in DMEM medium and cultured in 24-well plates at 37°C and 5% CO2. They were allowed to adhere for 24 h, at which point cells were labeled with 4 µM PKH26. We prepared MFC cells that were either untreated or treated for different concentration of wogonin (50, 100 and 200 µM) for 2 hours. After MoDCs washed with cold PBS for three times, MFC cells labeled with 0.5 µM CFSE were added into 24-well plates for phagocytosis assays. After 15 min at 37°C, phagocytosis was observed by Leica DFC 450C software, using the ImageJ1.38 image processing and analysis software calculating the percentage of MoDCs that engulfed at least one tumor cell in at least 100 cells.

### Statistical analysis

The values in the figures are expressed as the means ± standard deviation (SD). The figures in this study were representatives of more than 3 different experiments. Statistical analysis of the data between the control and treated groups was performed by a student t test. Values of p<0.05 were considered as statistically significant.

## Results

### Wogonin induced calreticulin (CRT) translocation to cell surface membrane is dependent on the PERK and PI3K/AKT

**Figure 1:**
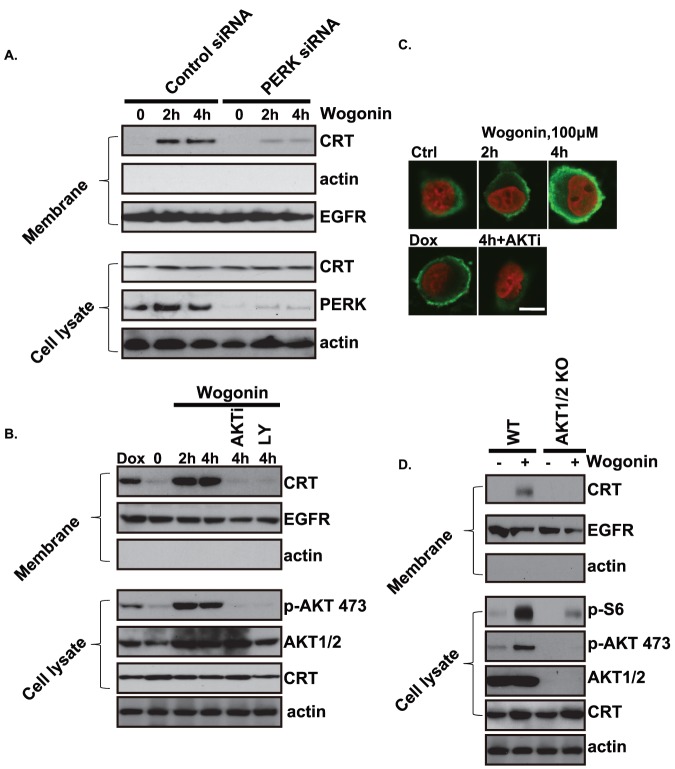
Wogonin induced calreticulin (CRT) translocation to cell surface membrane is dependent on the PERK and PI3K/AKT. Gastric carcinoma cell line MFC cells were transfected with scramble siRNA (Ctrl, 200 nM) or PERK siRNA (200 nM), after 48 hours, expression level of PERK was detected by Western blots to verify PERK level after siRNA treatment. Successfully PERK knockdown cells and their control cells (Ctrl siRNA treated cells) were treated with wogonin (100 µM) for 2 and 4 hours. Cell surface proteins in the plasma membrane fraction were than biotinylated and tested for CRT, EGFR and actin. Total cell lysate were also obtained to test CRT, PERK and actin (A). MFC cells were pretreated with AKT specific inhibitor X (AKTi 100 nM) or PI3K/AKT inhibitor LY294002 (100 nM) for 2 hours, followed by wogonin (100 µM) treated for 2 and 4 hours, CRT, EGFR and actin in the plasma membrane protein fraction were detected by Western blots. CRT, p-AKT (ser 473), AKT1/2 and actin in total cell lysate were also detected by Western blots (B).CRT translocation to cell surface after wogonin treatment was also confirmed by confocal immune-fluorescence microscopy, the translocation was inhibited by Akt inhibitor X (AKTi 100 nM), doxorubicin (Dox, 1 µM, 2 hrs treatment) was used here as positive controls. (C). WT and AKT1/2 double knockout MEFs were treated with wogonin (100 µM) for 4 hours; CRT, EGFR and actin in the plasma membrane protein fraction were detected by Western blots. P-S6 (S235/236), p-AKT (S473), AKT1/2, CRT and actin in whole cell lysate were detected by Western blots(D). Experiments in this figure were repeated at least 3 times and similar results were obtained. Bar = 10 µm.

Previous studies have shown that an external supply of signals that induce calreticulin (CRT) plasma membrane exposure, acting as an “eat me” signaling, which confers immunogenicity to otherwise nonimmunogenic cell death, allowing for an optimal anticancer chemotherapy [1]. Next we tested whether wogonin could also behave similarly in MFC cancer cells. As you can see in Fig. 1A, wogonin (100 µm) induced strong CRT cell surface translocation in MFC cancer cells in as detected by Western blots testing cell surface CRT. Among the signal pathways that regulates CRT translocation and corresponding immunogenic cell death/apoptosis, it is now well established that pre-apoptotic ER stress and the phosphorylation of the eukaryotic translation initiation factor 2α (eIF2α) and the upstream signal kinase protein kinase R-like endoplasmic reticulum kinase (PERK) are the main ones [13]. The phosphorylation of the eIF2α by the PERK anterograde transport of CRT from the ER to the Golgi apparatus and exocytosis of CRT-containing vesicles, finally results in CRT translocation onto the plasma membrane surface, which serves as key “eat-me” signal [1], [4], [14]–[17]. Here, we found that knockdown PERK by target siRNA largely inhibited CRT translocation by wogonin (Fig. 1A), indicating possible involvement of PERK and ER stress in this process. Surprisingly, PI3K/AKT inhibition by either pharmacological inhibitor (LY 294002 and AKT inhibitor X) or genetic knockdown (using AKT1/2 double knockout MEFs) also largely inhibited CRT translocation (Fig. 1B–D), the effect of AKT inhibition on wogonin induced CRT translocation was also confirmed by immune-fluorescence confocal assay as shown in Fig. 1C. Taken together, we found that wogonin induces CRT translocation to cell plasma surface, PERK and PI3K/AKT might be involved in this process.

### Wogonin induces ROS dependent ER stress response, severing as upstream signal for the activation of PI3K/AKT pathway

**Figure 2:**
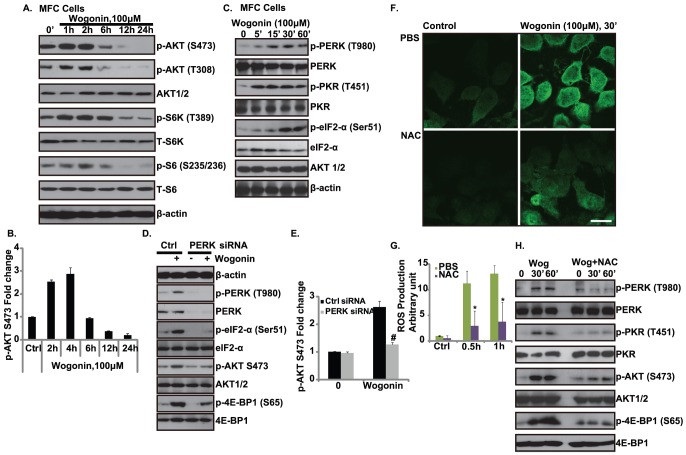
Wogonin induces ROS dependent ER stress response, severing as upstream signal for the activation of AKT. Gastric carcinoma cell line MFC cells were treated with wogonin (100 µM) for indicated time points, AKT and downstream mTORC1 activation were detected by western blots using indicated antibodies, AKT1/2, S6K, S6 and β-actin were also tested as equal loadings (A). AKT phosphorylation was quantified using Image J software after normalized to AKT1/2 (B). Note that Wogonin induced an early activation but later inhibition of AKT. MFC cells were treated with wogonin (100 µM) for indicated time points, p-PERK (Thr980), p-eIF2-α (Ser51), p-PKR (Thr446/451), their corresponding non-phospho proteins and AKT1/2 were detected by Western blots (C). MFC cells were transfected with PERK siRNA for 48 hours, successfully transfected cells (confirmed by western blot) were treated with wogonin (100 µM) for 1 hour, p-PERK (Thr980), p-eIF2-α (Ser51), p-AKT (Ser 473), p-4E-BP1 (Ser 65), PERK, their corresponding non-phospho proteins and β-actin were detected by Western blots(D), AKT phosphorylation was quantified using Image J software after normalized to AKT1/2 (E). MFC Cells were pre-treated with anti-oxidant N-Acetyl-Cysteine (NAC, 400 µM) for 2 hour, followed by wogonin (100 µM) treatment for 30 minutes, ROS production were detected by both fluorescence (F) and FACS (G) assay respectively as described above. MFC Cells were pre-treated with anti-oxidant N-Acetyl-Cysteine (NAC, 400 µM) for 2 hour, followed by wogonin (100 µM) for 30 and 60 minutes, p-PERK (Thr980), p-PKR (Thr446/451), p-4E-BP1 (Ser 65), p-AKT (Ser 473) and their corresponding non-phospho proteins were detected by Western blots(H). Experiments in this figure were repeated at least 3 times and similar results were obtained. **^#^** *P*<0.05 vs. Control siRNA group. *P<0.05 vs. group without NAC pre-treatment. Bar = 10 µm.

As we discussed above, our current data suggest that that wogonin induced CRT translocation, and PERK and PI3K/AKT pathway may be involved in this process. Next we tried to dissect this signal pathway. As shown in Fig. 2A, wogonin (100 µM) induced an obvious PI3K/AKT activation in MFC cells in a short time (up to 2 hours); interestingly AKT activation was down-regulated after long-term exposure of wogonin (Fig. 2A and B). At the same time, exposure of wogonin also induced an obvious ER response as evidenced by the strong phosphorylation of ER stress proteins (PERK, PKR and eIF2α) in wogonin treated MFC cells (Fig. 2C). PERK siRNA knockdown, which has been shown to inhibit CRT translocation (Fig. 1A), largely inhibited wogonin induced phosphorylation of eIF2α and PI3K/AKT activation in MFC cells (Fig. 2D and E), indicating that PERK serves as the upstream signal for wogonin induced PI3K/AKT signaling. By trying to identify the upstream signal for wogonin induced ER stress and PERK phosphorylation, we found that significant ROS production (Fig. 2F and G), PERK/PKR phosphorylation and PI3K/AKT/mTORC1 activation triggered by wogonin were largely inhibited by anti-oxidant N-Acetyl-Cysteine (NAC) (Fig. 2G–H). Based on this, we propose that ROS modifies ER proteins and elicit ER stress response, which results in PERK/PKR mediating eIF2α phosphorylation and subsequent PI3K/AKT activation, which directs CRT translocation and a possible “eat-me” signal.

### DNA-PKcs, forms a complex with PERK, mediates AKT activation by Wogonin

**Figure 3:**
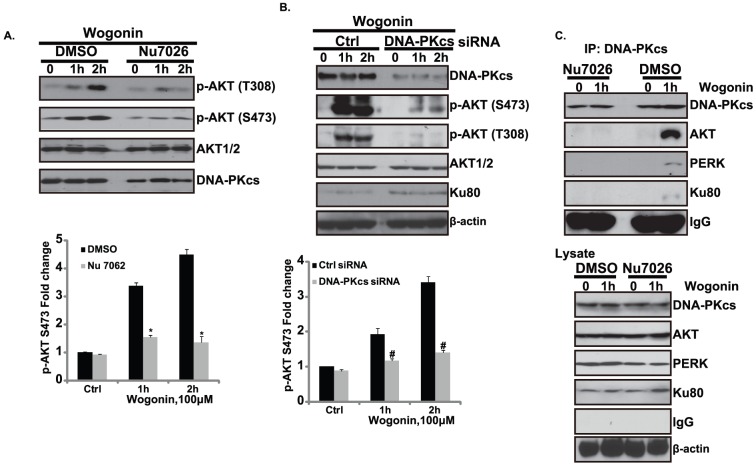
DNA-PKcs, forms a complex with PERK, mediates AKT activation by Wogonin. Gastric carcinoma cell line MFC Cells were pre-treated with a DNA-PKcs inhibitor (Nu 7062, 10 µM) for 2 hour, followed by wogonin (100 µM) treatment for 1 and 2 hours, AKT phosphorylation were detected by Western blot and DNA-PKcs and AKT1/2 were detected as equal loadings (A). MCF-7 cell were transfected with scramble (Ctrl, 200 nM) or DNA-PKcs (200 nM) for 48 hours by using transfection methods motioned above, successful transfected cells were used for testing AKT signaling in wogonin treated MFC cells (B). MFC Cells were pre-treated with a DNA-PKcs inhibitor (Nu 7062, 10 µM) for 2 hour, followed by wogonin (100 µM) treatment for 1 h, the precleared 600-µg aliquots of cell lysates were incubated with anti-DNA-PKcs, followed by Western blotting analysis with anti-DNA-PKcs, AKT, PERK, Ku80, IgG and β-actin respectively(C). Experiments in this figure were repeated at least 3 times and similar results were obtained. ^*^P<0.05 vs. group without Nu7062, ^*^P<0.05 vs. group without DNA-PKcs knockdown.

We next tried to indentify the intermediate player of AKT activation by PERK by focus on DNA-PKcs, a recently discovered PI3K member that can activate AKT [18]–[20]. DNA-PK is composed of a 470-kDa catalytic subunit (DNA-PKcs) and the Ku antigen complex (Ku80/Ku70) and involved in V(D)J recombination, repair of DNA double strand breaks by nonhomologous end joining, apoptosis, and transcription regulation [21].Significantly, now it is well established that DNA-PKcs, as a member of the PI3K family, also regulates AKT phosphorylation [22]. In addition, a role for DNA-PK in the activation of AKT by CpG-DNA has been established using bone marrow-derived macrophages [23]. Moreover, Bozulic et al. indicated that DNA-PK phosphorylates AKT upon induction of DNA double strand breaks [19]. Also, the requirement of DNA-PK to phosphorylates AKT in response to ionizing radiation was also established in vivo [19]. So next, we tested the possible involvement of DNA-PKcs in wogonin induced AKT phosphorylation. A small molecular inhibitor of DNA-PKcs, Nu7062, was specifically used here to inhibit DNA-PKcs activity. In cells pretreated with Nu7026, wogonin-induced AKT phosphorylation was almost totally abolished (Fig. 3A). These results supported our hypothesis that DNA-PKcs might be the key inter-mediates for wogonin induced AKT activation. To test this further, specific siRNA against DNA-PKcs was used here to knockdown DNA-PKcs. In full support of our data from the NU7026 studies, AKT phosphorylation on both Ser473 and Thr308 was largely impaired in wogonin treated cells depleted of DNA-PKcs (Fig. 3B). Importantly, we found that DNA-PKcs, AKT and PERK form a complex in Wogonin treated cells, which is reversed by pre-treatment with DNA-PKcs inhibitor Nu 7062 (Fig. 3C), based on this information, we suggest that DNA-PKcs, forms a complex with PERK, mediates AKT activation by wogonin. However, the detailed mechanism by which DNA-PKcs phosphorylates AKT in wogonin treated cells needs further investigation.

### Indentify Annexin A1 and p22 as possible targets of wogonin

**Figure 4:**
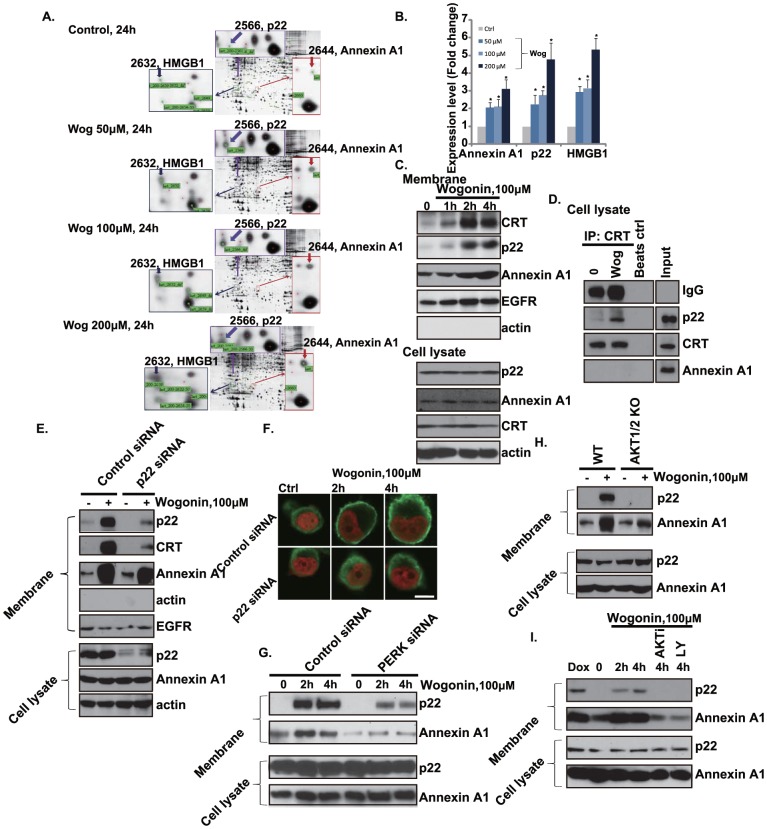
Indentify p22 and Annexin A1 as possible targets of Wogonin. Gastric carcinoma cell line MKN-45 cells were either left untreated or treated with wogonin 50, 100, 200 µM, respectively (A), Two-dimensional gel electrophoresis was performed and proteins were identified by mass spectrometry. Note a obvious induction of p22, Annexin A1 and HMGB1 after 24 hours, expression level of those proteins was quantified (B). MFC cells were treated with wogonin (100 µM) for 1, 2 and 4 hours, followed by immunoblot detection of CRT, p22, Annexin 1, EGFR and actin in the plasma membrane protein fraction and in whole cell lysate (C), pre-cleared 600-µg aliquots of cell lysates for the 4 hour time point were incubated with anti-CRT, followed by Western blotting analysis with anti-CRT, p22, Annexin A1 and IgG respectively (D). MFC cells transfected with control or p22 siRNA for 48 hours, p22 expression in whole cell lysate were detected by Western blot. Successful p22 knockdown cells were then treated with wogonin (100 µM) for 2 hours, followed by immunoblot detection of CRT, p22, Annexin A1, EGFR and actin in the plasma membrane protein fraction, p22, Annexin A1 and actin in cell lysate were also tested (E). The effect of p22 siRNA on CRT translocation was also confirmed by confocal immune-fluorescence microscopy assay(F). MCFs cells transfected with control or PERK siRNA (200 nM, for 48 hours) were treated with wogonin (100 µM) for 2 and 4 hours, followed by immunoblot detection of p22 and Annexin A1 in the plasma membrane protein fraction and in total cell lysate (G). WT and AKT1/2 double knockout MEFs were treated with wogonin (100 µM) for 4 hours, p22 and Annexin A1 in both plasma membrane protein fraction and total cell lysate were tested (H). MFC cells were pretreated with AKT specific inhibitor X (AKTi 100 nM) or PI3K/AKT inhibitor LY294002 (100 nM) for 2 hours, followed by wogonin (100 µM) treated for 2 and 4 hours, p22 and Annexin A1 in both plasma membrane protein fraction and total cell lysate were tested (I). ^*^P<0.05 vs. group without wogonin treatment.

To further indentify possible targets of wogonin, a two-dimensional (2D) gel electrophoresis plus mass spectrometry protein analysis was applied here. We prepared whole cell proteins from Gastric carcinoma cell line MKN-45 cells that were either untreated or treated for different concentration of wogonin (50, 100 and 200 µM) for 24 hours. Comparison of two-dimensional (2D) electrophoreses, followed by mass spectroscopic analyses, led to the identification of HMGB1 (high-mobility group protein 1), p22 and Annexin A1 as proteins that was strongly induced by wogonin treatment in a dose dependent manner (Fig. 4A and B). Annexin A1 is the first characterized member of the annexin family of proteins able to bind (i.e. to annex) to cellular membranes in a calcium-dependent manner. Originally described as a phospholipase A2 (PLA2)-inhibitory protein, Annexin A1 can affect many components of the inflammatory reaction besides the metabolism of arachidonic acid [24], [25]. Interestingly enough, Annexin A1 has been recently implicated in the apoptotic cell “eat me” signal and ensuing phagocytosis, and evidence is accumulating to support a role in the resolution phase of inflammation. A paper by Arur et al. elegantly demonstrated that Annexin A1 might serve as an endogenous phosphatidylserine (PS) ligand [26], mediating engulfment of apoptotic cells. Annexin A1 is recruited to the PS-rich region of cell surface and mediates “eat-me” signal and release cellular calcium [26]. Notably, we found that annexin A1 as well as calcium binding protein p22 were both exposure to cell surface after wogonin treatment as measured by Western blot testing cell surface proteins (Fig. 4C). P22, a widely expressed and evolutionary conserved Ca^2+^-binding protein, is among the less well characterized members of the EF-hand superfamily [10]. It is required for membrane traffic in a cell-free assay, which reconstitutes the targeting/docking/fusion of membrane vesicles with plasma membrane [27]. In this study we demonstrate that p22 also translocates to cell surface after wogonin treatment (Fig. 4C). Importantly, p22 siRNA knockdown largely inhibit wogonin induced CRT but not Annexin A1 translocation in MFC cells (Fig. 4E and F). By using immune-percipitation assay, we demonstrated that p22 associates with CRT but not Annexin A1 in wogonin treated cells, undergoing cell surface exposure (Fig. 4D). The results indicated that p22 may play an important role in CRT intracellular transition and cell membrane exposure. To further test possible upstream signal for this process, we found that inhibition PERK or PI3K/ATK diminished p22 and Annexin A1 cell surface exposure, indicating PERK/PI3K/AKT signal pathway mediates p22 and Annexin A1 cell surface exposure, further inducing “eat-me” signal(Fig. 4G–I).

### The HMGB1 and ATP release induced by wogonin and subsequent enhanced phagocytosis activities by activating dendritic cells

**Figure 5:**
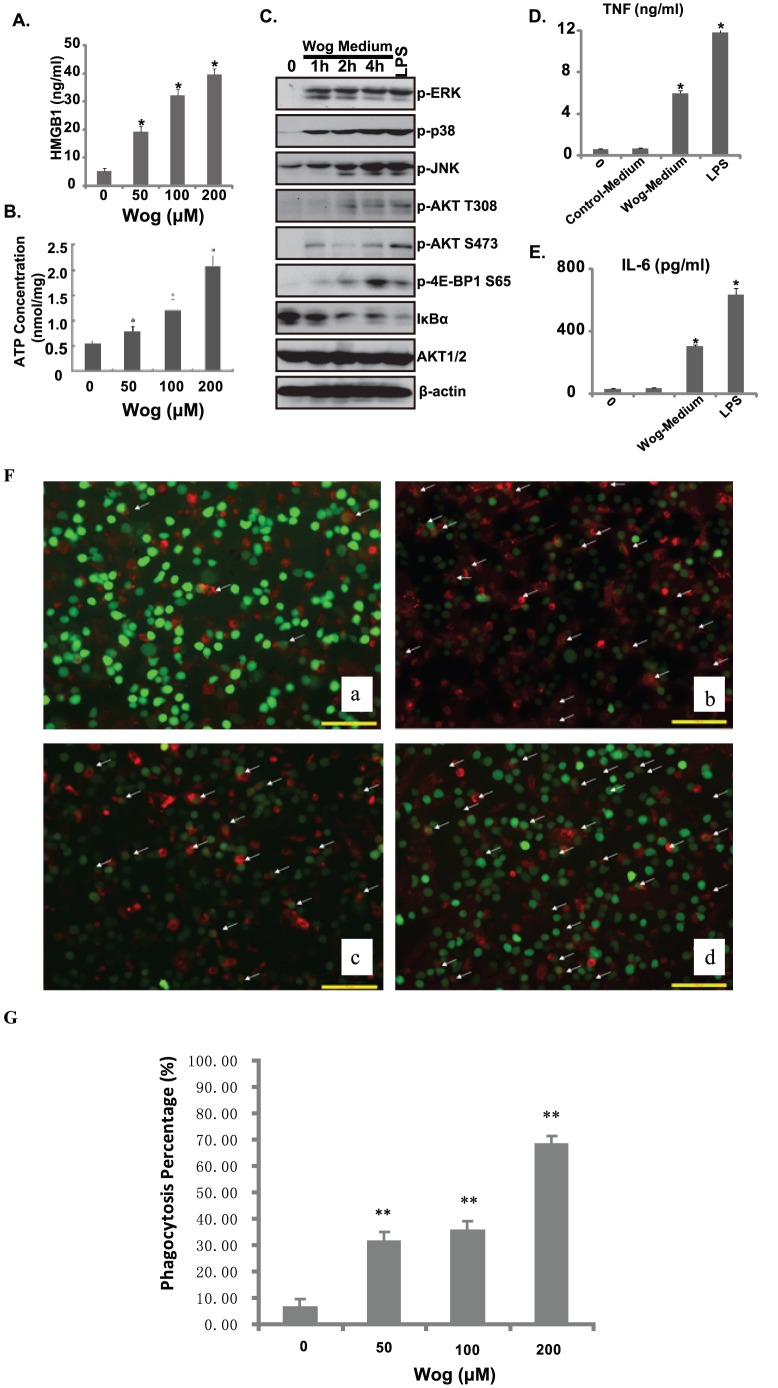
Wogonin induces the release of HMGB1 and ATP and activation of dendritic cells (DCs). Gastric carcinoma cell line MFC cells were treated with indicated concentration of wogonin (0, 50, 100 and 200 µM) for 36 hours, medium were collected and HMGB1 release (A) was detected by the ELISA assay and eATP was measured by Molecular Probes' ATP Determination Kit (B). Medium of MFC cells with or without 24 hours of wogonin (100 µM) treatment was then transferred to MoDCs, its signaling activation (PI3K/AKT/mTOR/MAPK/NF-kB) were detected by Western blots using indicated antibodies(C), TNF-α and IL-6 release from MoDCs were detected by ELISA assay 24 hours after indicated medium treatment (D and E). To detect the changes of phagocytic activities by treatment with wogonin, MoDCs were incubated with MFC cells in 0.1% DMSO or wogonin with 50 µM, 100 µM, 200 µM wogonin, respectively (Fa, b, c, d and G). Experiments in this figure were repeated at least 3 times and similar results were obtained. *P<0.05 and **P<0.01 vs. group without wogonin treatment.

HMGB1 is released into the extracellular environment during cell death. Extracellular HMGB1 “alarms” the innate immune system by acting as a chemoattractant for inflammatory leukocytes, functioning as an immune adjuvant for soluble and particulate antigens, and triggering activation of DCs [28], [29]. In addition, activated monocytes, macrophages and dendritic cells also secrete HMGB1, forming a positive feedback loop that results in the release of additional cytokines and neutrophils [30], [31]. Here, as evidenced by 2D-Gel and ELISA assay, wogonin induced HMGB1 expression (Fig. 4A) and release (Fig. 5A). Moreover; we find that wogonin significantly increased the release of extracellular ATP (eATP) (Fig. 5B). Released HMGB1 and ATP, together with other factors, activated DCs by activating PI3K/AKT/mTOR, MAPK and NF-κB signaling pathway (Fig. 5C) and induces cytokine (TNF-α and IL-6) release (Fig. 5D and E).

In order to detect the different phagocytosis of wogonin-treated and untreated MFC cells by MoDCs, MFC cells were labeled with CFSE green fluorescence and MoDCs were labeled with PKH26 red fluorescence. MoDCs that had engulfed MFC cells could be clearly distinguished from that had not engulfed cells. As shown in Fig. 5F and 5G, wogonin treated MFC cells could stimulate the phagocytic activity of MoDCs in a concentration dependent manner compared with that untreated with wogonin. The data suggest wogonin-treated tumor cell could elicit an inflammatory response in vitro, which might be involved in immunogenic cancer cell death. We suggested that it may be due to induction of HMGB1 and ATP release together with calreticulin and/or Annexin A1 translocation to the MFC cells surface by wogonin [32].

### Wogonin enhances the anti-tumor immunity of tumor cell vaccination in vivo

**Figure 6:**
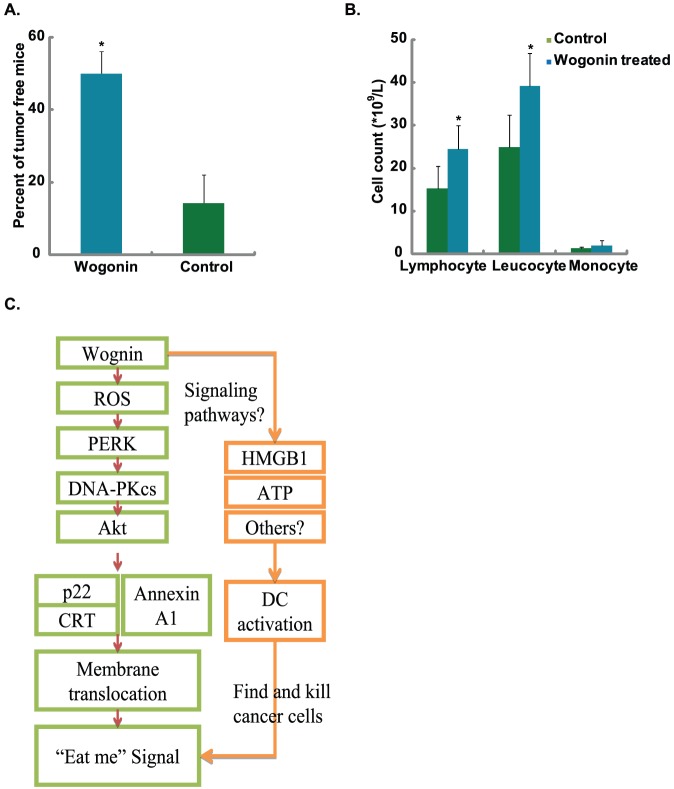
In vivo anti-tumor cell vaccination experiment. 3×10^6^ MFC cells, suspended in 200 ml PBS, either left untreaed or treated with wogonin (100 µM) for 4 h. Wogonin treated MFC cells were inoculated subcutaneously into the lower flank of 615 mice, whereas 5×10^5^ untreated control cells were inoculated into the contralateral flank 7 days later. Two weeks later, the tumor free mice were calculated (A) and the numbers of lymphocytes, leucocytes and monocytes in the blood were measured by flow cytometry (B). Experiments in this figure were repeated at least 3 times and similar results were obtained. n = 20. *P<0.05 vs. group without wogonin treatment. Hypothetical scheme of the signaling pathway for wogonin-induced immunity enhancement of cancer cell vaccine. Wogonin induces ROS production, which elicits ER stress response, the phosphorylation of ER stress proteins PERK and/or PKR activates downstream signal AKT in a DNA-PKcs dependent manner, mediates CRT/Annexin A1/p22 translocation and/or HMGB1/ATP release, acting as an “eat me” signaling and conferring immunogenicity(C).

In anti-tumor vaccination experiment, wogonin pretreated MFC cells can elicit significant antitumor response in 615 mice as described in previous study [1]. The rates of tumor free mice in wogonin pretreated MFC cells group and untreated group are 50% and 14%, respectively (Fig. 6A), indicating wogonin pretreatment significantly enhances the inhibition effects of tumor cell vaccine on homoplastic grafted gastric tumor growth in mice. We also found that the numbers of lymphocytes, leucocytes and monocytes in the blood of mice in wogonin pretreated MFC cells group were significantly higher than that in untreated group mice (Fig. 6B). This suggests DCs mediated cytotoxicity T lymphocyte (CTL) activation might be involved. The above results confirmed that the immunogenicity of wogonin pretreated tumor cell vaccine could be identified and presented by DCs, subsequently an immunogenic cancer cell death occurred due to CTL infusion and an inflammatory response *in vivo*.

## Discussion

Previous studies have shown that an external supply of signals that induce CRT and Annexin A1 plasma membrane exposure, acting as an “eat me” signaling and allowing for an optimal anticancer chemotherapy [1], [26]. In this study, we find that wogonin also induces CRT/Annexin A1 membrane exposure (Fig. 1) as well as the release of HMGB1 and ATP (Fig. 4 and 5), which might play an important role in antitumor immunity. Then the underlying signaling pathway was identified. We propose that wogonin induces ROS production, which modifies ER proteins and elicit ER stress response, the phosphorylation of ER stress proteins PERK/PKR activates downstream signals including eIF2α and AKT, which promotes CRT/Annexin A1and HMGB1 translation (Fig. 2 and 3). Thus the requirements of PERK/AKT in wogonin induced immunity enhancement of tumor cell vaccine are identified. The in vivo tumor cell vaccine experiment confirms this conclusion.

We observed that PI3K/AKT activation was increased by short-term exposure to wogonin but was down-regulated by long-term exposure (Fig. 2A). ER stress-induced AKT activation has been reported by a few other groups [13], [33]–[35]. Thus, it is possible that in response to wogonin, cells quickly activate AKT, which serves as a key intermediate for ER stress induced “eat me” signal including CRT/P22/Annexin A1 exposure and/or HMGB1/ATP release. On the other hand, when wogonin exposure is prolonged, and down-regulated Akt activation occurs, leading to cell death. The mechanism by which long-term wogonin exposure or other ER stress leads AKT inactivation needs further study. Indeed, our and other groups have recently observed that ER stress induced long-term deactivation of AKT and caused cell death [36]–[38]. The conclusion that PERK is involved in wogonin induced PI3K/AKT activation is consisted with previous study, which found that induction of the PI3K/AKT pathway was impaired in PKR(−/−) or PERK(−/−) MEFs in response to various ER stress inducers [13], [33]–[35]. However, the exact mechanism for PERK/PKR activating AKT remains unclear. Antonis E. Koromilas and his colleagues suggested that the most conceivable explanation is that inhibition of protein synthesis by eIF2α phosphorylation blocks the synthesis of a protein(s) that negatively regulates PI3K activity [34]. In this current study, we discovered that DNA-PKcs, a member of PI3K family and recently discovered upstream signal for AKT, is required for wogonin induced AKT activation. DNA-PKcs forms a complex with PERK, mediating AKT activation in wogonin treated cells (Fig. 3).

Recently, Kodi S. Ravichandran et al. identified ATP and UTP as a critical and non-redundant “find me” signal released by apoptotic cells [39]. ATP in the immune system provides a costimulatory signal to T cells and driving the differentiation of T helper 17 (TH17) cells mediated by a subpopulation of DCs [40], [41]. The larger increases in eATP that are associated with cell death serve as a key “danger” signal in inflammatory processes by activation of P2X7 receptors, which leads to the processing and release of interleukin 1β (IL-1β) [42], [43]. Another aspect of the ATP-dependent inflammatory response is chemotaxis. ATP exerts a direct chemotactic effect on eosinophils, neutrophils, and immature (but not mature) DCs [44]. This type of communication thus detects the process of cell death and leads to an inflammatory response and, potentially, to kill the living tumor cells. Here we found that wogonin induces ATP release in a doses dependant manner (Fig. 5B), which might be the key “eat me” signal. However, the detailed mechanism by which wogonin induces ATP release needs further investigation.

In conclusion, wogonin can enhance the immunity of tumor cell vaccine, in which ER stress activated AKT signaling pathway mediated calreticulin/Annexin A1 translocation and/or HMGB1/ATP release are involved (Fig. 6C). The challenges for future investigation are to identify the role of Annexin A1 in chemotherapies or ionizing irradiation induced potent anti-tumor cell vaccine.
